# A New Type of COP: The Role of Circulating Osteoprogenitor (COP) Cells in Health and Disease

**DOI:** 10.1093/geroni/igab046.167

**Published:** 2021-12-17

**Authors:** Gustavo Duque

## Abstract

Circulating osteogenic progenitor (COP) cells are a population of cells in the peripheral blood with the capacity for bone formation and broader differentiation into mesoderm-like cells in vitro. While some of their biological characteristics are documented in vitro, their role in the aging process and the pathogenesis of musculoskeletal diseases remains yet to be thoroughly evaluated. This translational session will go from bench to bedside, reviewing the current evidence on COP cells. In this session, we will provide an overview of the role of COP cells in the aging process and a number of physiological and pathological conditions and identify areas for future research. In addition, we will suggest possible areas for clinical utilization in the management of musculoskeletal diseases, which include novel diagnostic and therapeutic uses.

